# Altemeier operation associated with dynamic graciloplasty: a case report

**DOI:** 10.1186/1752-1947-3-9317

**Published:** 2009-12-04

**Authors:** Massimo Mongardini, Roberto Paolo Iachetta, Alessandra Cola, Eleonora Degli Effetti, Filippo Custureri

**Affiliations:** 1 Department of Surgical Sciences, Division of General Surgery L, "Sapienza" University of Rome, Italy

## Abstract

**Introduction:**

More than 80% of patients with full-thickness rectal prolapse have co-existing fecal incontinence. Choosing the ideal surgical strategy is always a difficult task. We combined an Altemeier rectosigmoid resection with anal dynamic graciloplasty to provide a functional neosphincter. We found no published reports describing this surgical association.

**Case presentation:**

We report the case of a 72-year-old Caucasian woman with full-thickness rectal prolapse associated with fecal incontinence from severe neuromuscular damage.

**Conclusion:**

Combined dynamic graciloplasty and an Altemeier operation could be a valid therapeutic option in patients with severe rectal prolapse with fecal incontinence from severe neurogenic damage.

## Introduction

More than 80% of patients with full-thickness rectal prolapse have co-existing fecal incontinence [1]. The physiopathology of this condition remains partly unknown. According to recent studies, ultrasonography documents a lesion involving the internal or external anal sphincter or both in 71% of patients, while in the remaining 29% incontinence arose from marked anorectal sphincter complex weakness related to severe pudendal neuropathy or to excessive internal sphincter inhibition secondary to the prolapse-associated chronic stimulation of the inhibitory anorectal reflex [2]. Choosing the ideal surgical strategy for managing prolapse-associated fecal incontinence is always a difficult task.

In a patient who presented recently with full-thickness rectal prolapse associated with fecal incontinence from severe neuromuscular damage, we combined an Altemeier rectosigmoid resection with anal dynamic graciloplasty to provide a functional neosphincter. This combined procedure has the advantage of avoiding the risk that correcting the rectal prolapse alone might lead to the removal of the terminal obstacle, namely the rectosigmoid intussusception, and thus worsening fecal incontinence.

## Case presentation

We present the case of a 72-year-old Caucasian woman with a history of childhood encephalitis with motor sequelae, who presented with a 10-year history of full-thickness rectal prolapse that had progressively worsened despite two surgical procedures, namely, anal encirclement 13 years before presentation and a new encirclement associated with stapler mucous prolapsectomy 6 years before presentation. For 2 years, severe fecal incontinence associated with repeated rectal bleeding had prevented her from sitting down, had severely impeded her walking and induced pain. The patient's Wexner incontinence score was 19, and anorectal manometry showed marked hypotonia of the anal canal at rest (20 mmHg) and during contraction (40 mmHg). Endorectal ultrasonographic examination revealed no documentable sphincter lesions although the muscle fibers appeared markedly thinned. Electromyographic (EMG) recordings disclosed severe neurogenic damage to her external anal sphincter. The patient declined to undergo construction of a definitive colostomy.

The operation proceeded in three steps. First, the full-thickness rectal wall was incised circumferentially at 2 cm from the pectinate line. The pouch of Douglas was opened and about 20 cm of bowel was prepared before the peritoneal fossa was reconstructed. Once the bowel was resected a coloanal anastomosis was constructed with a 29 circular stapler. The operation proceeded with dynamic graciloplasty. Through two longitudinal incisions on the medial face of the right thigh, the gracile muscle was mobilized down to its insertion on the tibial tuberosity. Once the muscle was prepared for tunneling, electrical stimuli were delivered to identify the neurovascular peduncle. This step is crucial to identify the site for definitive intramuscular electrode implantation that guarantees an effective gracilis muscle contraction.

Second, the gracile muscle was tunnelled and wrapped around the sigmoid colon anastomosed to the residual rectum after preparing the peri-anastomotic space using two longitudinal perianal incisions. This fixed the muscle tendon on the perineal skin.

Finally, a subcutaneous pouch was created in the right iliac fossa to house the neurostimulator. The leads connecting the neurostimulator to the gracile muscle were then tunnelled subcutaneously. This entailed constructing a temporary transverse colostomy to minimize the risk of infections involving the perianal accesses that can damage the neosphincter or cause its disinsertion.

The patient had an uneventful postoperative course, and on day 7 began regular leg gymnastics with a soft balloon placed between her knees. Neurostimulation delivered at low frequency began on day 20 and continued for about 2 months before the frequency was increased. In the sixth month, clinical examination and manometric evaluation showed a slight improvement in sphincter tone, that is, pressure at 30 mmHg without electrical stimulation and 55 mmHg with electrical stimulation. One year after the operation, the colostomy was closed under manometric evaluation (pressure at 40 mmHg without electrical stimulation and 65 mmHg with electrical stimulation).

Two years after the combined operation, no further recurrent rectal prolapse was visible. The patient was already continent for solids (Wexner incontinence score 9) and could switch the pacemaker device on and off without help.

## Discussion

We found no published reports describing the combined dynamic graciloplasty and Altemeier operation we used to treat this patient who had rectal prolapse associated with fecal incontinence. Although this association is a relatively common problem in older individuals, patients presenting with this socially distressing disorder are often severely debilitated and have often undergone various treatments that provided no meaningful results. It is thus important to select an individual management strategy from among the various therapeutic options that will improve fecal incontinence and improve the patient's quality of life.

The cause of our patient's complete rectal prolapse was unclear. Although its complex pathophysiology remains incompletely understood, major known causative factors include abnormalities of the pelvic floor, rapid reduction in adipose tissue in the ischiorectal fossa (an important factor in children) and obstructed defecation and psychic disturbance especially in older patients.

In this case, as in about 80% of known cases [1], the patient had full-thickness rectal prolapse with co-existing fecal incontinence, that is, involuntary excretion of fecal material at inappropriate moments or places recurring more than twice a month [2]. We attributed this problem to the pudenda - nerve damage seen on the external sphincter EMG. Pudendal neuropathy is among the main causes of this association, as well as organic damage to the external sphincter, for example after obstetric or surgical anorectal trauma, and prolapse-associated altered stimulation of the anorectal inhibitory reflex.

Because no reference therapeutic standard exists for managing full-thickness rectal prolapsed, especially in patients with co-existing fecal incontinence, in managing our patient's problems we had to select the surgical procedure most likely to repair the rectal prolapse, diminish fecal incontinence, and improve her quality of life. Numerous surgical procedures, including abdominal and perineal approaches, exist for managing rectal prolapse [3,4].

In an older, debilitated person such as the patient in our case, in whom all other treatments was proven ineffective, the most indicated perineal operation is the Altemeier procedure (rectosigmoidectomy), currently combined with anterior levatorplasty. Because this combined technique uses the transanal approach, it has the advantage of being relatively non-invasive. It can also be done rapidly (55 to 120 minutes) and leads to low intraoperative blood loss (45 to 180 ml) [3]. The disadvantages, however, are high recurrence rates, which reach 58%, with a mean value around 6 to 10% depending on the length of follow-up [3,5,6] and, because the Altemeier procedure leaves pre-existing fecal incontinence unchanged, a high incidence of postoperative incontinence (22% to 56% of cases) [6,7].

Because the rectal prolapse in our patient co-existed with fecal incontinence for liquids and solids and EMG evidence showed abnormal sphincter function related to pudendal neuropathy, after an Altemeier resection alone this condition would probably have persisted or even worsened. Combining the Altemeier procedure with dynamic graciloplasty therefore proved an appropriate choice because it circumvented these problems.

The use of the nearby gracile muscle to reconstruct the anal sphincter was first described in 1952 [8]. The clinical results of dynamic graciloplasty were later improved by implanting a pacemaker device to stimulate the gracile muscle electrically. The first reported dynamic graciloplasty dates back to 1991 [9,10]. Dynamic graciloplasty is indicated in the treatment of severe fecal incontinence caused by irreparable organic sphincter damage from irreversible neurogenic pudendal nerve damage or congenital disorders. It can also be used for anorectal repair after a Miles abdominoperineal resection. The long-term aim of chronic gracile muscle neurostimulation is to replace voluntary contraction and exert a sustained contraction that transforms fatigue-prone (type II) muscle fibers into the fatigue-resistant (type I) fibers that physiologically account for 80% of the external sphincter [11]. Electrical stimulation of the neosphincter elicits a forceful tonic contraction yielding basal anal pressures from 56 to 95 mmHg as assessed by anal manometry. When the patient uses the pulse generator to turn the stimulator off, the neosphincter relaxes thus allowing evacuation.

The improved outcome in fecal continence for solids in this patient at 2 years after combined surgery receives confirmation from multiple studies in patients treated with dynamic graciloplasty alone [12,13]. The success rate is at an average of 60% [3]. Although our patient had none of the reported early complications, including infections and pain in the lower limbs, the possibilities of long-term complications like stimulator malfunction, remains [2].

Of the various therapeutic options available to surgically repair rectal prolapse associated with fecal incontinence, combining the two operations seemed a valid choice in this older, debilitated patient. We considered an artificial anal sphincter an unfeasible option, given the problems in surgical management related to our patient's advanced age and motor deficits and, equally important, the high rates of infection (about 20%) [14]. Given the EMG findings of severe sphincter denervation, we could not have used sacral nerve neuromodulation, which is an undoubtedly valid technique with a promising future for patients whose incontinence is resistant to conservative measures [15].

## Conclusion

Co-existing full-thickness rectal prolapse and fecal incontinence is an anorectal disorder that is hard to manage. The ideal therapeutic choice depends on numerous factors, such as the patient's general condition and local disease, and the surgeon's expertise in using available surgical techniques. These variables make it difficult to standardize an operative procedure. The combined dynamic graciloplasty and Altemeier operation we propose could be a valid therapeutic option in patients with severe rectal prolapse with fecal incontinence from irreversible neurogenic damage.

## Consent

Written informed consent was obtained from the patient for publication of this case report and any accompanying images. A copy of the written consent is available for review by the Editor-in-Chief of this journal.

## Competing interests

The authors declare that they have no competing interests.

## Authors' contributions

MM and FC analyzed and interpreted the patient's medical data, made the surgical plan and performed the operation. RPI was a major contributor in writing the manuscript and also participated in the surgical operation. AC also contributed in writing the manuscript and participated in the surgical operation. EDE wrote the literature review. All authors read and approved the final manuscript.
